# Effectiveness of a network of automatically activated trained volunteers on the reduction of cardiopulmonary resuscitation manoueuvers initiation time: study protocol

**DOI:** 10.1186/s12889-019-6896-9

**Published:** 2019-05-14

**Authors:** Albert Del Pozo, Felipe Villalobos, Cristina Rey-Reñones, Ester Granado, David Sabaté, Carme Poblet, Angels Calvet, Josep Basora, Antoni Castro, Gemma Flores

**Affiliations:** 1 Research Group in Primary Care Research Technologies (TICS-AP), Primary Care Research Institute IDIAP Jordi Gol, Reus, Spain; 2 Primary Care Centre Falset. Primary Care Management Camp de Tarragona, Catalan Institute of Health, Tarragona, Spain; 3 Research Support Unit Camp de Tarragona, Primary Care Research Institute IDIAP Jordi Gol, Camí de Riudoms 53-55, 43202 Reus, Tarragona Spain; 4 University Rovira i Virgili, Tarragona, Spain; 5 Primary Care Centre Reus-4, Primary Care Management Camp de Tarragona. Catalan Institute of Health, Tarragona, Spain; 6 Primary Care Centre CUAP Reus, Primary Care Management Camp de Tarragona, Catalan Institute of Health, Tarragona, Spain; 7 Mobility Office, Catalan Institute of Health, Barcelona, Spain; 8 Open University of Catalonia, Barcelona, Spain; 9 Internal Medicine Department, Sant Joan de Reus University Hospital, Reus, Spain; 10 Analysis and Quality Unit, Health and Social Network Santa Tecla, Tarragona, Spain

**Keywords:** Out-of-hospital cardiac arrest, Cardiopulmonary resuscitation, Automated external defibrillators, Emergency medical system services, Mobile phone, Technology, Primary healthcare

## Abstract

**Background:**

Cardiorespiratory arrest (CRA) is a health emergency with high mortality. Mortality depends on time of initiation and quality of cardiopulmonary resuscitation (CPR) manoeuvres and the use of the automated external defibrillator (AED).

**Methods:**

The aim of the study is to determine the effectiveness of an automatically activated network of volunteers using smartwatch and smartphone applications on the reduction of time of initiation of cardiopulmonary resuscitation manoeuvres. The protocol will be developed in four phases: 1) validation of an application (App) for smartwatch that automatically generates a health alert in case of out-of-hospital cardiorespiratory arrest (OHCA); 2) training course for laypersons on CPR manoeuvres and AED use; 3) creation of a network of volunteers trained in CPR and AED use that covers our city; and 4) simulation in which the network of volunteers is automatically activated via smartphone to attend simulated OHCAs. A total of 134 health alerts will be generated; on 67 occasions the alert will be directed to the emergency health services and to the network of trained volunteers (Intervention Group) and on 67 occasions the alert will be solely directed to the emergency health services (Control Group). The arrival time of the first rescuer, category of first rescuer (emergency services versus network of volunteers), initiation time of manoeuvres and competence will be recorded.

**Discussion:**

CPR training for laypersons is advised, especially for relatives and people close to patients with heart disease, to reduce time of initiation of CPR and to improve OHCA survival rates. This study aims to verify that the initiation time of CPR manoeuvres and AED use is shorter in the intervention than in the control group.

**Trial registration:**

Clinicaltrials.gov ID NCT03828305. Trial registered on February 1, 2019 (retrospective register).

## Background

Sudden death due to cardiorespiratory arrest (CRA) causes more than 30,000 deaths per year in Spain [1]. Many of these deaths occur in public spaces in the presence of other people. In these situations, the percentage of deaths exceeds 90%, and more than half of the survivors will present some neurological sequelae [2]. The chain of survival is a series of sequential actions aimed at increasing the survival of CRA victims. It consists of the following 5 steps [3]: a) early recognition and activation of emergency medical services (EMS); b) Immediate high quality cardiopulmonary resuscitation (CPR) by CRA witnesses; c) Rapid defibrillation by automated external defibrillator (AED); d) Basic and advanced emergency medical services; and e) post-CPR cures. Time of initiation of CPR manoeuvres by witnesses can double and even treble survival in out-of-hospital cardiorespiratory arrest (OHCA) cases (3). The main cause of OHCA is ventricular fibrillation (VF) and its treatment consists of defibrillation using an AED. Every minute of delay in the use of an AED reduces the chance of survival by 10–12% [4]. Studies show (ILCOR Level of Evidence I) that the interval between the EMS call and defibrillation must be less than 5 min [5]. In our region, the response interval between the call to EMS and their arrival is usually greater than 8 min [6].

### Training and evaluation of competencies in CPR

Any social group can receive CPR training. Since 70–85% of OHCA occur at home and 15–25% in public spaces, it is crucial to train the largest number of people [7] in CPR manoeuvres and AED use. The training plan should target two main population groups: a) Relatives who are close to patients at high risk of OHCA, since their probability of witnessing an OHCA is 66–75% [7]; and b) CPR training in schools (training of teachers who will subsequently train pupils).

### Mobile phones and CPR

Mobile phones are currently present in 95.6% Spanish homes, and 48.6% of these phones have GPS [8]. This could facilitate improvements in the chain of survival [9], since with these new technologies the EMS can be immediately alerted without the need to leave the victim, while simultaneously providing the victim’s location through the GPS. Several mobile applications (Apps) can assist people in emergency situations (Alpify.com, PulsePoint, Goupto.me, My112), but they require voluntary activation by the user.

A Smart City uses information and communication technologies to increase the efficiency of services offered to its citizens [10]. In a Smart City the infrastructures are equipped with the most advanced technological solutions to facilitate the interaction of the population with different institutional, urban and technological assets [10]. We aim to create a network of volunteers in our health region by means of an App. Previous experiences illustrate the effectiveness of networks of volunteer resuscitators. For instance, a study conducted in Stockholm showed that the use of the mobile phone to activate an alert and send it to the EMS and a network of volunteer resuscitators reduced initiation time of CPR manoeuvres by 2 min and 20 s [11]. In 51.1% cases, CPR was initiated before the arrival of EMS, which resulted in significantly higher survival rates than in the group in which the CPR manoeuvres were only initiated by EMS (10.5% vs. 4%, *p* < 0.001) [12]. A recent randomized clinical trial (RCT) also conducted in Stockholm with a network of volunteer resuscitators interconnected by mobile phone in which 667 OHCA were attended, it was observed that in 62% of the cases of the intervention group the CPR manoeuvres were initiated before the arrival of the ambulance, compared to 54% in the control group (*p* < 0.001) [13]. In these two cases, the medical alert was activated by witnesses.

This study underscores the possibility of automatically alerting the EMS in case of OHCA using non-invasive methods (smartwatch and smart wristbands). We believe that it is necessary to integrate this system into a network of CPR-trained volunteers.

### Study aim

This community trial aims to determine the effectiveness on time of initiation of CPR manoeuvres and AED use, of a network of CPR and AED trained volunteers connected through an application for smartwatch which automatically activates in case of out-of-hospital cardiorespiratory arrest (OHCA).

### Methods/design

The study will be carried out in 4 phases: 1) Validation of an App for smartwatch that automatically activates a network of volunteers trained in CPR manoeuvres and use of AED (Network-CPR) and the emergency services, when the user of the smartwatch suffers a OHCA; 2) Training in basic CPR and use of the AED in the general population; 3) Creation of a network of volunteers from the subjects trained (Network-CPR); 4) Assessment of the effectiveness of the automatic activation of the Network-CPR through an app for smartwatch on time of initiation of CPR manoeuvres and AED use; and evaluation of quality of performance of members of the Network-CPR through simulations around the city.

### Development of the mobile app

An App for smartwatch and another App for smartphone have been developed based on the Android operating system, and a platform has been installed in the central computer of the Primary Care Emergency Centre (PCEC). In the absence of heart rate (> 3 s without heartbeat) and when the smartwatch wearer falls down, the application sends an alert in the first place to the smartwatch so that the smartwatch wearer can deactivate it in case of false alarm. If it is a real alert and after 3 s the smartwatch wearer does not deactivate it, a warning alert is generated and sent automatically to the App installed on the smartphones of the Network-CPR members that are within a radius of 300 m from where the warning alert has been generated, and simultaneously to the platform installed in the PCEC central computer. The members of the Network-CPR will receive on their smartphone the signal with the geolocation of the OHCA, the map with the fastest route to reach the location of the OHCA (Google Maps), and the locations with available AEDs.

### Phase 1. Validation of the app

Research team will conduct the task as follows: (1) Check of automatic activation and precision of geolocation in various locations of the city, verifying that the alert reaches the members of the Network-CPR and the PCEC; (2) For a period of 3 months, four volunteers at high risk of CRA will wear the smartwatch to determine the likelihood of false activations (false positives).

### Phases 2 and 3. Selection and training of the network-CPR

The network will include population groups that are routinely in contact with people at risk of OHCA (police, firemen, teaching staff, health workers, shopkeepers, pharmacists, university students, gym instructors and others), as well as relatives of people with heart disease. In the case of elderly people living alone, neighbours and caregivers will be included. According to our records, 2105 people with heart disease susceptible to OHCA live currently in our city.

During a 6-month period prior to the courses, all health-care professionals of the primary care centers (PCC) from the city will invite adult users of the services to participate voluntarily in the training courses. Participants’ data (name and telephone number) will be directed to the research team. At the same time, the research team will contact to different population groups to invite them to training. Each participant will be contact by phone for a member of the research team to confirm the participation and schedule the date and time of the training. The protocol aims to train 430 people (20% relatives of people with heart disease).

The training courses on basic CPR manoeuvres and use of the AED will be in accordance with the protocol of the Catalan Council of Resuscitation (CCR) [14], and will be delivered by instructors accredited by the CCR. A total of 30 sessions will be organised, with groups of 8–10 people per instructor.

At the beginning of the training courses variables such as age, sex and social class (based on the British Registrar’s General Classification, which uses three classes: high (I-II), medium (IIIN-IIIM) and low (IV-V)) [15] will be collected.

At the end of the training courses, instructor will evaluate of competence of participants: a) check scene; b) assess level of consciousness; c) open airway; d) check spontaneous breathing; e) ask for help; f) chest compressions (quality); g) frequency of compressions; h) two ventilations (quality); i) alternate compressions/ventilations. Each item scores 1; in a correct CPR manoeuvre, 8 of the 9 items evaluated have been adequately performed [16].

At the end of the training courses, the research team will explain the project and the possibility of participation in the Network-CPR that will cover the city. Recruitment of 100 participants (approximately 25% of trained people) is expected. Criteria for participating are as follows: a qualification of suitable on the evaluation of competences during the course; age over 18 years; having a smartphone with Android operating system; signing the informed consent form; and agreeing to have the alerts’ App installed on the smartphone.

### Phase 4. Simulation study

A total of 134 situations will simulate CRA warning alerts in the city. On 67 occasions the alerts will reach the Network-CPR and the emergency services (Intervention Group) and on 67 occasions only the emergency services (Control Group). The warning alert will be randomly generated location-wise, based on the 51 locations where a CRA was notified to the emergency services during the year 2017. The intervention group will be notified with alerts that will arrive through the App for smartphones of the members of the Network-CPR that are in a radius of 300 m in the first instance and a radius of 600 m in the second instance, if no member was found in the closer radius; and through the platform installed in the PCEC central computer in the case of the emergency services. The control group will be notified by telephone calls to the PCEC, following the usual EMS protocol.

The simulations will be carried out morning and afternoon. During the time of the simulations a physician and an emergency nurse (PCEC team) will be in the PCEC waiting for the warning alert to go to the locations and perform the simulations.

At the locations there will be two trained health practitioners (Victim Team: physician or emergency nurse and an instructor accredited by the CCR), with a CPR mannequin and an AED Trainer.

### Measurements

During the simulations, the Victim Team will be responsible for creating the simulated alerts, will collect time-related variables and assess the competences of the Network-CPR.

1. Initiation time of simulated OHCA (T0). Time (hour: minutes: seconds) when the simulated alert is created and sent.
2. Initiation time of manoeuvres (Tim): time (hour: minutes: seconds) elapsed between initiation time of simulated OHCA (T0) and initiation time of CPR manoeuvres (T2b or T3b in case of arrival of PCEC team or Network-CPR, respectively). It is considered that the manoeuvres begin when the chest compressions are started in the manikin.
3. Initiation time of AED use (Tid): time elapsed between initiation time of simulated OHCA (T0) and time of AED use (T2d or T3d in case of arrival of the PCEC team or Network-CPR, respectively). It is considered that the use of the AED starts when the patches are placed on the thorax of the manikin.
4. Response time of the PCEC team (Trc): time between initiation time of simulated OHCA (T0) and arrival of the PCEC team (T2).
5. Response time of the Network-CPR (Trr): time between initiation time of simulated OHCA (T0) and arrival of the Network-CPR (T3).
6. AED arrival time (TllD): Time elapsed between initiation time of simulated OHCA (T0) and arrival of the AED (T2c or T3c, in case of arrival of PCEC team or Network-CPR, respectively). It is necessary to register who brings the AED, members of the Network-CPR, PCEC staff or an external person to whom assistance has been requested.
7. It must be recorded whether these manoeuvres are initiated by members of the PCEC-team or Network-CPR.
8. Evaluation of competence of Network-CPR members, based on the same evaluated criteria during the training courses.

### Sample size/power calculation

The sample size calculation was based in the minimum response time between mobile responder and emergency medical as the main dependent variable, and using the following criteria: a power of 80% and a significance level (alpha risk) of 5%, assuming error-simulations of 10%, a common standard deviation of 4.3 units and a difference ≥ 2.2 units (in reference to the Ringh et al. study realised in Stockholm [13]), 67 simulations per group (intervention and control) will be needed. The estimated sample size was calculated using the Granmo software (version 7.13; Granmo; IMIM Hospital del Mar, Barcelona, Spain).

### Statistical analysis

Mean and standard deviation will be used to describe quantitative variables if they follow a normal distribution, otherwise median and interquartile range will be used. The Kolgomorov test will be used to assess the normality of the variables. The qualitative variables will be described in percentages and 95% confidence intervals. Student’s *t*-test will be used to analyse the differences between start times of CPR manoeuvres. Linear Regression Models adjusted for potentially confusing variables will be used for multivariate analysis.

### Ethics approval and consent to participate

The study will follow the tenets of the Declaration of Helsinki [17], and Good Clinical Practice guidelines. The protocol has been approved by the Clinical Research Ethics Committee of the Primary Care Research Institute IDIAP Jordi Gol. Data confidentiality will be protected under the Spanish Organic Law of Personal Data Protection 15/1999, December 13. All participants are required to sign the informed consent form.

### Data storage and dissemination

All data from the study will be stored in a secure database, stripped of identifiers. Methods for data management and coding can be obtained by contacting the corresponding author. The results of the study will be disseminated through publications, reports, and conference presentations.

## Discussion

This article describes the protocol of a study that aims to determine the effectiveness on start time of CPR manoeuvres and AED use of a network of trained volunteers connected through a smartwatch App which automatically activates in case of out-of-hospital cardiorespiratory arrest (OHCA).

This study is instrumental for the validation of a smartwatch connected to an application for smartphone and the PCEC central computer, which automatically activates the first link in the survival chain (early recognition of CRA and activation of emergency services). The automation of this first step will reduce start times of CPR manoeuvres and AED use, thus increasing survival rates and improving the quality of life of patients.

In addition, this study could serve as health promoter by training laypersons in CPR and AED use. It is also a stepping stone towards smart health in the context of a smart city, in which new technologies facilitate more efficient communication between the population and health professionals.

## Abbreviations

AED: Automated External Defibrillator
CPR: Cardiopulmonary Resuscitation
CRA: Cardiorespiratory Arrest
EMS: Emergency Medical Services
GPS: Global Positioning System
OHCA: Out-of-Hospital Cardio-respiratory Arrest
